# From Ewald sphere to Ewald shell in nonlinear optics

**DOI:** 10.1038/srep29365

**Published:** 2016-07-08

**Authors:** Huang Huang, Cheng-Ping Huang, Chao Zhang, Xu-Hao Hong, Xue-Jin Zhang, Yi-Qiang Qin, Yong-Yuan Zhu

**Affiliations:** 1 Key Laboratory of Modern Acoustics, National Laboratory of Solid State Microstructures, and National Center of Microstructures and Quantum Manipulation, Nanjing University, Nanjing 210093, China; 2 Department of Applied Physics, Nanjing Tech University, Nanjing 210009, China

## Abstract

Ewald sphere is a simple vector scheme to depict the X-ray Bragg diffraction in a crystal. A similar method, known as the nonlinear Ewald sphere, was employed to illustrate optical frequency conversion processes. We extend the nonlinear Ewald sphere to the Ewald shell construction. With the Ewald shell, a variety of quasi-phase-matching (QPM) effects, such as the collective envelope effect associated with multiple QPM resonances, the enhanced second- harmonic generation due to multiple reciprocal vectors etc., are suggested theoretically and verified experimentally. By rotating the nonlinear photonic crystal sample, the dynamic evolution of these QPM effects has also been observed, which agreed well with the Ewald shell model.

Stemming from the X-ray diffraction, the Ewald sphere is a convenient method to depict the Bragg diffraction in a crystal, where the diffraction resonance arises when a reciprocal lattice point is located on the Ewald sphere^1^. The Ewald construction was also employed for the photonic crystals to study the light diffraction properties, which reveals the structural symmetry of artificial crystals or enhances light extraction from the light emitting diodes^2^,^3^. The Ewald sphere may be extended to the nonlinear optics as well. In 1998, Berger proposed the concept of nonlinear photonic crystal (NPC)^4^, which owns a homogeneous dielectric constant and a modulated nonlinear coefficient. For 1D NPC such as the domain-inverted ferroelectric crystals, QPM frequency conversion usually occurs in the collinear geometry^5^,^6^,^7^ with exceptional early works on the non-collinear cases, e.g., I. Freund’s study of nonlinear diffraction^8^. For the 2D counterpart, however, non-collinear QPM (or nonlinear diffraction) and multiple QPM processes become popular because of more reciprocal lattice points provided^4^,^9^,^10^,^11^. To describe the non-collinear effect geometrically, nonlinear Ewald sphere construction was proposed^4^, which indicates that, if a point of reciprocal lattice intersects the nonlinear Ewald sphere, a QPM resonance will be resulted. The method is also useful for studying some other nonlinear effects^12^,^13^,^14^,^15^,^16^, such as the scattering-assisted conical second-harmonic (SH) generation, the nonlinear Cherenkov radiation, etc.

Here, we extend the concept of nonlinear Ewald sphere to the nonlinear Ewald shell. As we know, the non-collinear QPM effect relies on the dimensions of Ewald sphere and reciprocal lattice. If the period of NPC is rather small and the reciprocal lattice points distribute sparsely, there will be few or discrete QPM resonances. But given a dense distribution of reciprocal points, more QPM processes may come into being and thus present new effects. Following this idea, a collective envelope effect because of multiple QPM resonances has been observed and analysed with the Ewald shell. An enhanced SH due to the simultaneous participation of multiple reciprocal vectors has also been suggested. Moreover, the dynamic evolution of these effects was experimentally studied, which matches well with the Ewald shell model.

## Methods

We chose a z-cut congruent LiTaO_3_ 2D NPC with rectangular lattice as our study subject. These samples are 0.5 mm thick, having periods of 12.15 × 12.61 μm along the x and y directions, and a total length of 5 mm along both directions. We polished two x-surfaces of the sample. A z-polarized 1064 nm Nd: YAG laser beam of 5 ns pulse width and 10 Hz repetition rate was chosen as the fundamental wave (FW). The output FW intensity was controlled by a half-wave plate and a Glan-laser polarizer. Then the FW was loosely focused with a waist-width of 0.5 mm and directed onto the centre of one polished x-surface of the sample. The sample was secured on a rotating multi-dimensional adjustment platform, and it was encaged in a black box. We aligned the rotating axis onto the vertical bisector of the sample’s input x-surface and put a projection plane 5 cm away from the far end of the sample to collect the output 532 nm SHs. The black box we used to encage the sample has two openings, one on the input end, and the other the output end. The input end opening is wide-open to ensure that FW could penetrate the sample while we rotate the sample with respect to the z-axis (also the optic axis of the sample) with an arbitrary angle of interest. The output end of the box has a small cut-slit exact the size of the output end of the sample, and we fix our sample onto that slit to secure that only lights from that output face of the sample could reach the projection screen freely. The experiment was conducted under room temperature in a darkroom. The schematic view of the experimental setup is shown in the Supplementary materials.

**Figure 1:**
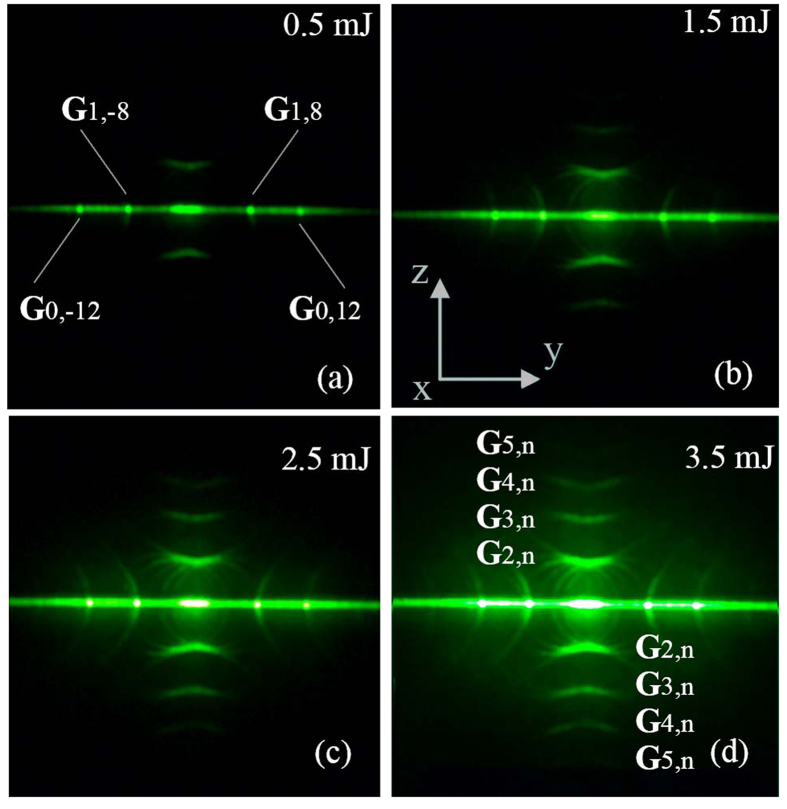
Scattering-assisted QPM and their envelopes with perpendicularly incident FW. Values of input power are given out on the corresponding image. Z-axis is also the sample’s optic axis and the polarization of the FW. FW comes in along the x-axis.

In the experiments, we fixed the NPC and let the FW perpendicularly beam onto the input surface. The FW input power was raised gradually from zero to 3.5 mJ per pulse. Figure 1 shows the observed results. Generally, the SHs on the projected screen include a horizontally widespread SH line and some symmetrically distributed SH arcs. The horizontal SH line is a random phase-matching effect due to period inaccuracy after electric poling^17^,^18^. We focus our deliberations on those SH arcs. With the input FW intensity as low as around 0.5 mJ, there was only one pair of concave and convex SH curves in the upper and lower space of the horizontal SH line. As the intensity going up, a series of similar curves occurred in succession, filling the even higher and lower space of the black background. And the dimly casting rings between the curve pair indicated these pairing curves were actually the envelopes of a series of scattering-assisted QPM conical SH projection^16^.

## Discussions & Conclusions

**Figure 2:**
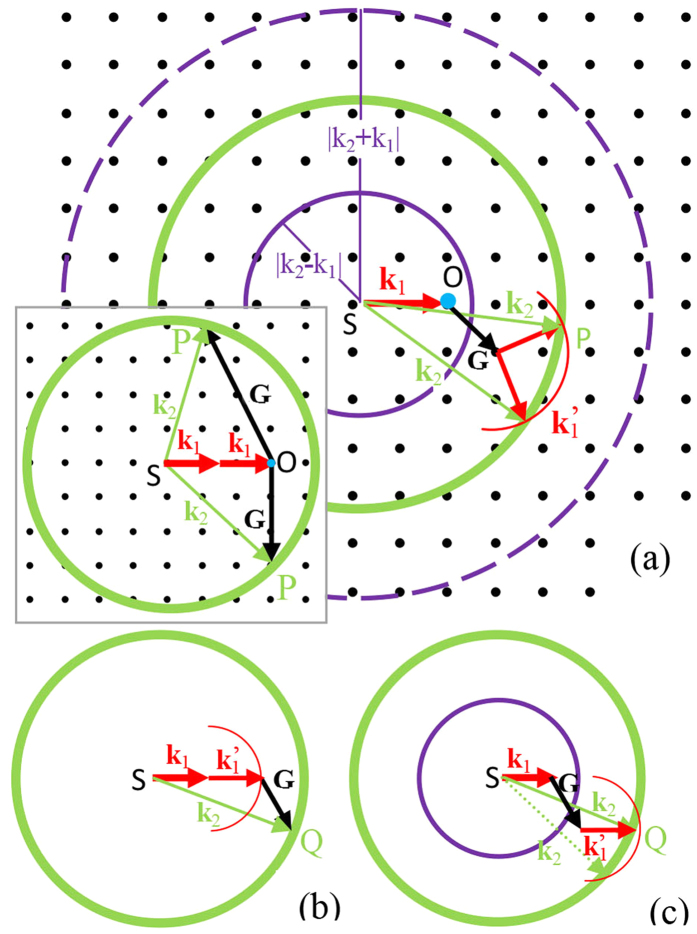
Introduction to Ewald shell scheme. (**a**) Ewald shell method in the scattering-assisted QPM process. Inset shows the nonlinear Ewald sphere construction; any RLV **G** residing on the Ewald sphere can form a QPM process, which outputs in **SP** direction. (**b**) Non-collinear QPM process in the Ewald sphere diagram. (**c**) Same non-collinear QPM process as (**b**) in Ewald shell diagram. The green arrow with dotted line indicates potential invisibility due to large output angle. All red half circles represent the scattering FW wave vectors. The blue dot on the arrow head of **k**_1_ indicates the origin of the reciprocal space.

Now we propose the Ewald shell method in Fig. 2(a), which was inspired by the nonlinear Ewald sphere method^4^ (more details can be found in the Supplementary materials). The aim of constructing the Ewald shell is to visualize the overall picture of the scattering-assisted QPM. First we attach the tip of wave vector **k**_1_ to the origin of the reciprocal space (O). Then we draw three spheres with their radii being |k_2_ + k_1_|, k_2_ and |k_2_ − k_1_|, respectively. In this procedure, we make sure these spheres co-centre at the starting point of **k**_1_ (S). Any reciprocal lattice vector (RLV) **G** with its tip within and on the *shell* (we call this shell as the nonlinear Ewald shell) enclosed by the largest and smallest sphere may represent a possible conical beam as the RLV **G** should fulfil the vector relationship k_1_ + k_1_′+ G = k_2_. That is, the wave vectors of the SH **k**_2_, the FW **k**_1_ and **k**_1_′ can join an enclosed vector polygon with a selective RLV **G** (**k**_1_′ originates from the elastic scattering of the FW^12^. In the NPC, small defects inside the crystal and tiny refractive-index modulation around the domain walls may cause a scattering of the FW. Nonetheless, for the practical NPC, the scattering sources are largely random and the directivity effect of scattering is very weak. The scattered light spreads elastically: the wave vectors of scattered FW are diffused in a small cone with the frequency remaining unchanged.). Thus, a conical SH beam may form, its output direction is decided by all **k**_2_ that passes through the intersection of the **k**_2_ sphere with a scattering vector **k**_1_′ sphere. When actually applying the Ewald shell, one may focus on the dynamics of the conical beams without drawing out all those conical beams, as we could show later in this article that analysing the RLVs in and on the Ewald shell is enough to reveal this dynamics. And since every RLVs in the Ewald shell may be related to infitesimal possbile scattered FW to form an SH, the Ewald shell method may inspire its user to create or deconstruct complex QPM processes with different RLVs.

We limit our discussion of the Ewald shell method with RLVs restricted within the *shell* region between the inner two spheres, namely, those with radii of k_2_ and |k_2_-k_1_|, respectively: The large sphere with radius of |k_2_ + k_1_| always relates to large angle scattering, extreme high-order RLVs or backscattering phase matching phenomena^19^, some of which are still short of experimental evidences at the moment.

With this backup, we can easily deduce that the first-shown curve pairs under 0.5 mJ FW input power was actually the envelopes of the scattering-assisted QPM conical beams with RLV series **G**_2,n_ (n = 0, ±1, ±2). And the subsequent acquaintances of the curve pairs under higher pump power are envelopes of conical beams related to a series of RLV **G**_3,n_, **G**_4,n_, etc. We tried to obtain the maximal magnitude of **G**_m,n_ corresponding to the detectable envelopes of scattering-assisted QPM conical beams by using total reflection angles as a rough restriction, which limits the scalar value of this maximum **G**_m,n_ as


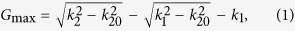


where k_20_ is the wave vector of the SH in vacuum. The corresponding maximal m of **G**_m,n_ is m_max_ = Floor[G_max_/k_1_] in our case. The actual number of the detectable output SH beams can be smaller due to intensity depletion of the large-angle-scattered FWs^12^ or just a low FW input power. Following Eq.(1), m_max_ with our sample should be 12, yet we only detected envelopes to an order of 7 by naked eye. We didn’t present conical beams or their envelopes of **G**_m,n_ with order m > 5 in Fig. 1, as the samples are easily damaged under powerful input.

Besides this envelope effect, non-collinear QPM processes could be traced. Even under a low FW intensity, two pairs of phase-matching dots (RLV **G**_0, ±12_ and **G**_1, ±8_ involved) called our attention in the horizontal SH line. With the FW power rising, outward SH arcs appeared passing through these non-collinear QPM spots. Why the coincidence?

As mentioned earlier, conventional QPM processes (with the collinear or *non-collinear* geometry) can be viewed as special cases of the scattering-assisted QPM processes. Figure 2(b) shows a nonlinear Ewald sphere diagram of the vector matching in a non-collinear QPM process. By rearranging the vector composition of Fig. 2(b) and taking elastic scattering of the FW into account, we put all the same vectors into the Ewald shell diagram (Fig. 2(c)). We notice that the spot Q on **k**_2_ sphere where the conventional QPM occurs, actually locates a scattering vector **k**_1_′ sphere intersecting with the **k**_2_ sphere. The passing through of the intersecting circle on that spot indicates a scattering-assisted QPM process, or a conical beam. This suggests that the conventional non-collinear QPM processes are simply the conical beams restricted in the 2D crystal plane, and any non-collinear QPM process should have output components out of the crystal plane.

All of the above was convinced by our experiment. But as a general category of QPM, practices with the scattering-assisted QPM can be more complicated than that has just been discussed, even restricted in geometrical perspective. Below we’d like to share one particular.

**Figure 3:**
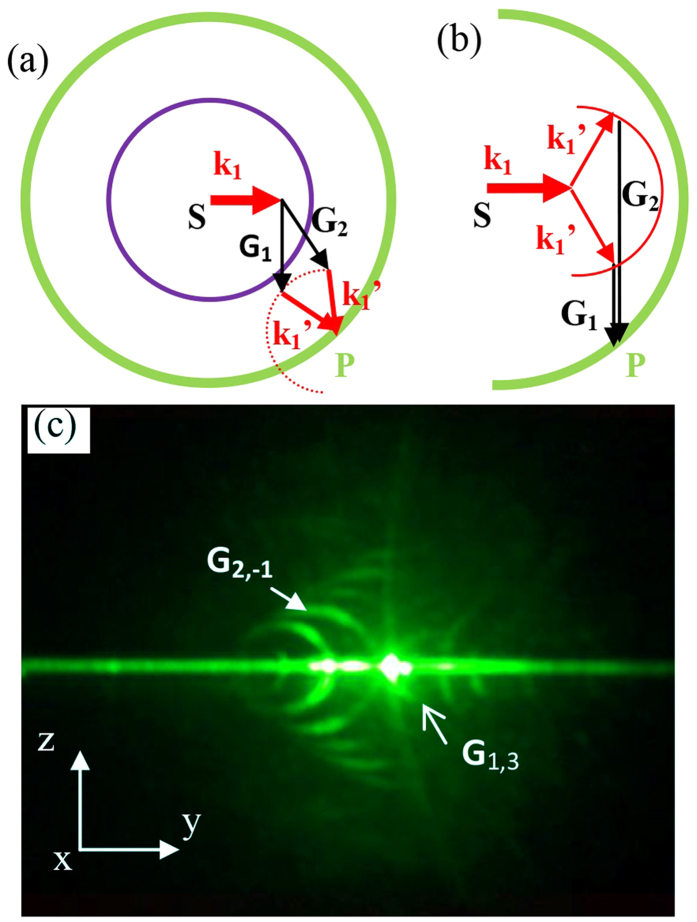
QPM processes occur with multi-RLV simultaneous coupling. (**a**) General 2D situation with Ewald shell diagram shows that two RLV **G**_1_ and **G**_2_ could simultaneously form a SH output in **SP** direction. (**b**) Similar interpretation of the nonlinear Ewald sphere method with scattering-FW considered. (**c**) An experimental example. The sample rotated 23° from the initial perpendicularly incident position with respect to the z-axis. Note that the background was lit up in this situation.

The scattering mechanism greatly expands the possible phase-matching modes and potentiates one single input FW to utilize more than one RLV to achieve QPM processes *at the same time*. By this, we mean that two or more explicitly different RLVs can simultaneously take part in the nonlinear coupling process, not just two or several conventional QPM processes occurring in tandem or in series^10^. We present more delicacy of our argument in Fig. 3. This seems a quite peculiar situation, as it’s very picky to find the underpinning RLVs, if the regular RLVs of NPC is not much crowded in the Ewald shell. As we know, in the X-ray diffraction, only a few diffraction peaks can be obtained in certain directions. By rotating the crystal and reciprocal lattice, the reciprocal lattice points may intersect the Ewald sphere, thus leading to the new diffraction spots. Here, if we rotate the NPC sample, and thus its reciprocal space, then the purpose of letting several RLVs play in concert might be rightly served.

And here goes the sample rotation with respect to the FW as the second part of our experiment. We fixed the incident spot still at the centre of the input surface. The rotational axis was through this spot and parallel to the optic axis. And the FW input power was remained at 1.2 mJ per pulse. During the rotation, conical beams and their envelopes deformed. Usually when the part of the conical beam in the sample plane happened to be a non-collinear QPM and the scattering angle of the phase-matching FW is in the vicinity of zero, the intensity of the conical beam would be enhanced. However, it is the moment that several elastic scattering conical beams coincided at one spot that the most powerful output occurred (Fig. 3(c)). Surely this cannot be just the attribute of the conventional non-collinear QPM process, as one can see that by reviewing Fig. 2 that one conventional non-collinear QPM process corresponds to just one conical beam.

**Figure 4:**
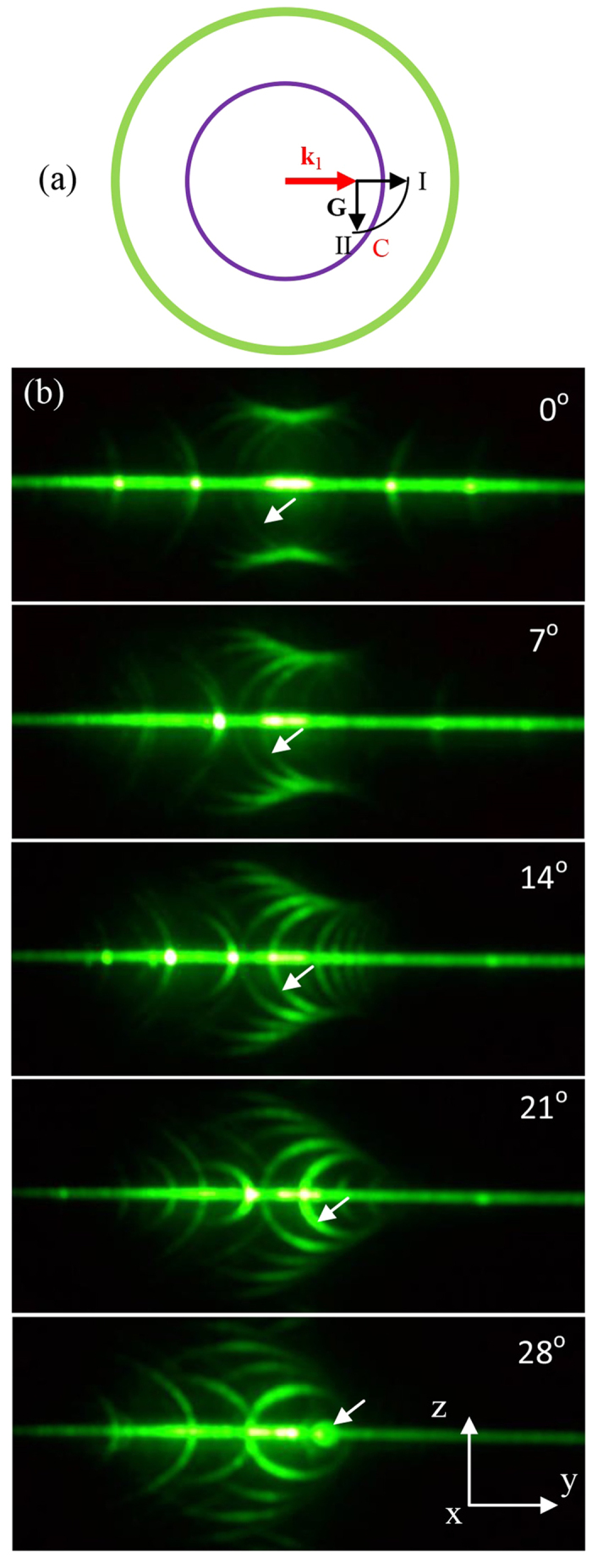
Annihilation/creation of the elastic scattering conical beams during sample rotation. (**a**) A vector **G** undergoes a rotation from phase matchable configuration I to phase unmatchable configuration II. (**b**) The annihilation of a conical beam with RLV **G**_2,2_; little white arrows reveal the exact positions of the corresponding conical beam.

Perhaps one more thing is noteworthy in this rotational dynamics. During the course, some of the phase-matchable RLVs may become phase unmatchable, yet some phase unmatchable matchable. This makes a rotation outcome quite animated by observing the annihilation and creation of the conical beams. This annihilation/ creation dynamics have primarily two bases, besides total internal reflection. Firstly, the NPC structure and hereby its reciprocal space are rigid. And the Ewald shell picture of the phase-matching lacks a complete spherical rotational symmetry as the origin of the reciprocal space O and the centre of the Ewald shell S do not coincide, but with a distance of one wave number k_1_. Figure 4(a) shows one possible situation. When the Ewald shell is rotating, the configuration can be changed from I via C to II. With the configuration I, the RLV **G** is phase-matchable, and there is a corresponding conical beam showing up. But with configuration II, the vector **G**, same RLV undergone a rotation along with the sample, is now phase unmatchable that the corresponding conical beam could not materialize. So any conical beam associated with **G**_m,n_ will be created/annihilated by rotating an angle of θ, which satisfies


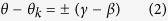


where *θ*_*k*_ is the rotating angle of **k**_1_ when the sample rotates θ, γ is the original angle between **G**_m,n_ and **k**_1_, and


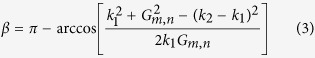


is the angle between **G**_m,n_ and **k**_1_. With our initial condition of perpendicular incidence, Eq.(2) can be further reduced to


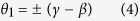


where *θ*_1_ = arcsin[sin *θ/n1* is the refraction angle of k_1_, and n_1_ is the refractive index of the FW in the NPC. If it’s a creation process, the “+” sign is applicable in both Eqs (2 and 4); otherwise, use the “−” sign. Some of our results are shown in Table 1.

Now we focus our attention to just one RLV **G**_2,2_ and its corresponding conical beam in our experiment (Fig. 4(b)). Numerical prediction suggested this conical beam would disappear at a rotational angle of 26.6° from the perpendicular-input position. The experiment agrees with the prediction. As the rotation angle increases, the radius of the conical beam shrinks gradually. With the sample rotation, reaching up to 28°, we observed that the projected conical beam with this RLV evolves from a ring locating almost at the very centre of the captured image to a degenerated spot, and then finally vanished.

The Ewald shell method can be applied to other nonlinear processes. For instance, it predicts the existence of elastic backscattering conical SH beams. Sum-frequency conical beam is also one, yet with additional complex, as there should be two Ewald shells to deal with the individual scattering process of each input. We shall leave further discussions in the future.

## Additional Information

**How to cite this article**: Huang, H. *et al*. From Ewald sphere to Ewald shell in nonlinear optics. *Sci. Rep.* **6**, 29365; doi: 10.1038/srep29365 (2016).

## Supplementary Material

Supplementary Information

## Figures and Tables

**Table 1:** RLV rotating angles to reach annihilation/creation configuration.

RLV	G_2,2_	G_2,3_	G_2,4_	G_1,4_	G_1,3_
Annihilation/Creation (A/C)	A	A	A	C	C
Theory^a^ (Deg.)	26.6	22.4	21.3	9.7	19.5
Experiment (Deg.)	28	24	22	10	20

^a^Following Eq. (4).
