# Supplementary material for: From Ewald sphere to Ewald shell in nonlinear optics
Source: Sci Rep. 2016 Jul 8;6:29365. doi: 10.1038/srep29365 (PMC4937392; doi:10.1038/srep29365)
Supplement: Supplementary Information [file srep29365-s1.pdf]

From Ewald sphere to Ewald shell in nonlinear optics

Huang Huang, Cheng-Ping Huang, Chao Zhang, Xu-Hao Hong, Xue-Jin Zhang, Yi-Qiang Qin & Yong-Yuan Zhu*

Supplementary Materials

This document provides further discussions about some details that haven't been covered in the main text of the article, due to the overall word limit of the ***Scientific Reports***.

Contents

Note on the construction of the Ewald shell
Deduction of Eq. (1)
Deduction of Eq. (2) & (3)
Experimental setup
Real-time Ewald shell example

Note on the construction of the Ewald shell

The second harmonic (SH) waves, the fundamental wave (FW) and its scattered waves interact in the nonlinear photonic crystal (NPC). We decided not to preclude any possibility of the output direction of the SH and the scattered direction of FW until theoretically and experimentally proven otherwise. Thus, the set of the output SH can be presented as a vector ball with all possible $\mathbf{k}_2$ included (Fig. S1(a)). So can be the set of scattered FW with all possible $\mathbf{k}_1$' (Fig. S1(c)). In order to maintain a simple illustration, usually we use the envelop sphere of the vector balls to represent them, where Fig. S1(b) is related to the $\mathbf{k}_2$ ball, and Fig. S1(d) is related to the $\mathbf{k}_1$' ball. Here, to construct the basic scheme of Ewald shell, we redraw the scattered wave vector $\mathbf{k}_1$' ball by reversing all the $\mathbf{k}_1$' vectors' directions (Fig. S1(e)), then we set it on the $\mathbf{k}_2$ envelop sphere with an explicitly drawn-out $\mathbf{k}_2$ (Fig. S1(f)), and the $\mathbf{k}_1$' ball centre is at the tip of that specific $\mathbf{k}_2$. And we draw out the FW $\mathbf{k}_1$. Obviously, any Reciprocal lattice vector (RLV) $\mathbf{G}$ with its tail on the tip of $\mathbf{k}_1$ and its tip on the sphere of $\mathbf{k}_1$' could fulfil the vector equation $\mathbf{k}_1 + \mathbf{k}_1' + \mathbf{G} = \mathbf{k}_2$, that means each one of these RLVs can form a conical beam. Here, the explicitly-shown $\mathbf{k}_2$ just indicates one of the possible SH directions in a conical beam.

Please take an immediate note that when doing this, we have implied that the origin of the reciprocal space does not coincide with the centre of the vector ball $\mathbf{k}_2$.

Now if we want to acquire the complete set of RLVs relating to all the possible output $\mathbf{k}_2$, the only thing we have to do is move the $\mathbf{k}_1$' ball throughout the $\mathbf{k}_2$ sphere surface (Fig. S1(g)). Then it becomes clear that the suitable RLVs are in the shell between the two spheres, which are with diameter of $|k_2 - k_1|$ and $|k_2 + k_1|$,

respectively. And again, the centre of this shell is not the origin O of the reciprocal space. So the reciprocal space does not have a spherical symmetry in the Ewald shell construction.

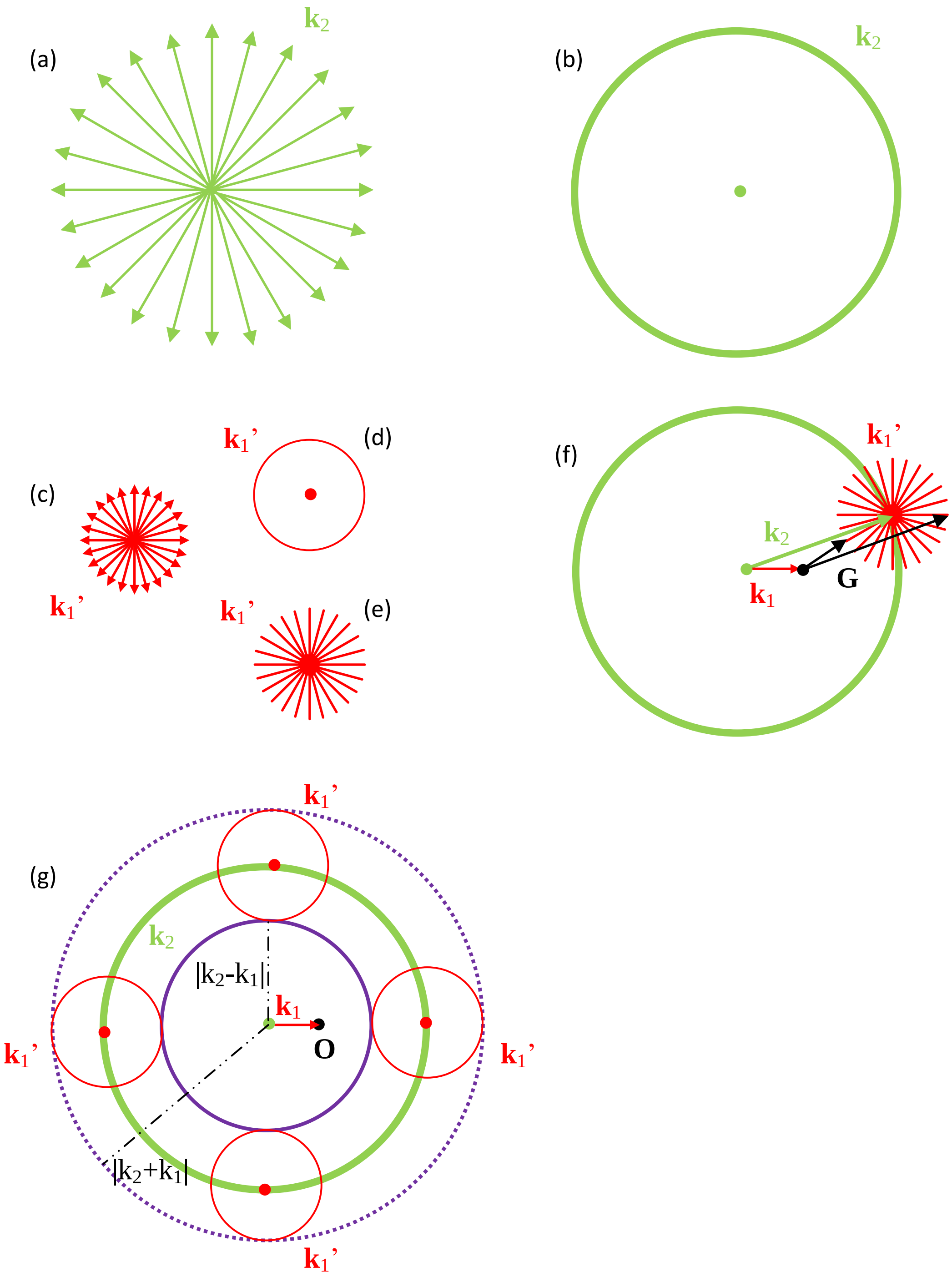

FIG. S1: The construction of the Ewald shell. (a) Possible output SH wave vectors can form a vector $\mathbf{k}_2$ ball. (b) The outer sphere envelop of the $\mathbf{k}_2$ ball; the green round dot inside is the centre of the sphere. (c) The scattered FW wave vectors $\mathbf{k}_1$' could also form a vector ball. (d) The outer sphere envelop of the $\mathbf{k}_1$' ball; the sphere centre is also represented by the red round dot. (e) The vector ball formed with the exact $\mathbf{k}_1$' ball by reversing all of the original vectors' directions. (f) A quasi phase-matching diagram represented with the $\mathbf{k}_2$ sphere. (g) Ewald shell construction with the point O as the origin of the reciprocal space with no RLVs explicitly drawn out.

Deduction of Eq. (1)

Eq. (1) is a rough criterion to decide the maximal detectable SH envelope arcs. Each index m of RLVs $\mathbf{G}_{m,n}$ is related to one of the horizontal SH envelope arcs. Thus, if we could decide the maximal value of index m for any possible $\mathbf{G}_{m,n}$ participating the scattering, we could get the maximal number of the output SH envelopes. And in all $\mathbf{G}_{m,n}$ of the same index m, $\mathbf{G}_{m,0}$ is the easiest one to analyse, as it's in the highest symmetrical position. And the conical beam related to $\mathbf{G}_{m,0}$ has the smallest output angle with a perpendicularly incident FW, which means if $\mathbf{G}_{m,0}$ related SH conical beam is not detectable, neither does other $\mathbf{G}_{m,n}$ related SH conical beams with the same index m.

So, now we present a symmetrical y-cut of the Ewald shell (Fig. S2), which includes $\mathbf{k_1}$ and the z-axis (also the optic axis) in this plane. The critical angle $\theta_C = Cos^{-1}\frac{1}{n_2}$ restricts the possible output SH, where n_2 is the refractive index of the SH. And we assume the maximal output SH is at the critical angle with the drawn-out wave vector $\mathbf{k}_2$ in the diagram to find out the $\mathbf{G}_{m,0}$ with the largest index m, namely $\mathbf{G}_{max}$. The scattered FW always has a wave number of k_1. If we draw a circular arc with its circular centre at the tip of the $\mathbf{k}_2$ and a diameter of k_1, the scattered FW $\mathbf{k}_1$' related to this $\mathbf{k}_2$ can be determined by the intersection of the arc and the x-axis (in the direction of $\mathbf{k}_1$) of the reciprocal space. We immediately acquire a vector triangle enclosed by $\mathbf{k}_2$, $\mathbf{k}_1+\mathbf{G}_{max}$ and $\mathbf{k}_1$'. With certain trigonometry and simplification, one can reach Eq. (1).

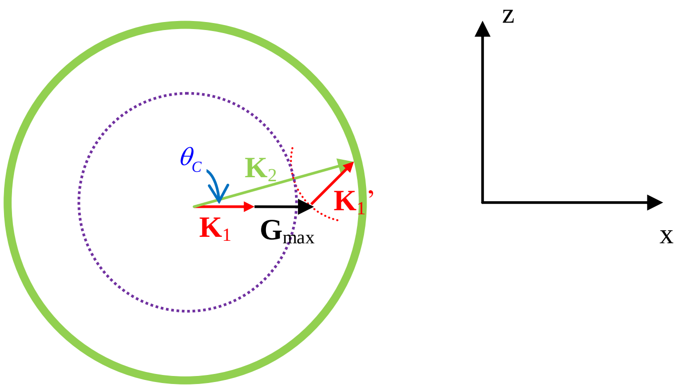

FIG. S2: A y-cut of the Ewald shell. $\mathbf{k}_1$ is along the x-axis, which is parallel to the $\mathbf{G}_{m,0}$ and thus $\mathbf{G}_{max}$. Between $\mathbf{k}_2$ and $\mathbf{k}_1$ there is the critical angle θ_C. The dotted red arc, whose intersection with the x-axis and the tip of $\mathbf{k}_1$ defines the $\mathbf{G}_{max}$, is actually part of the $\mathbf{k}_1$' sphere.

Deduction of Eq. (2) & (3)

Eq. (2) is a geometrical connection of the related vectors. Here we demonstrate the deduction of the Eq. (2) with an annihilation process. The counterpart of the creation process can be obtained similarly.

When studying the rotational dynamics of the conical beams, one may notice that the FW would rotates a smaller angle θ_k when the sample rotates an angle of θ, while the reciprocal space rotates an equal angle of θ during the process. And the angle β between RLV $\mathbf{G}_{m,n}$ and FW wave vector $\mathbf{k}_1$ at the critical configuration of $\mathbf{G}_{m,n}$ is a fixed value (Fig. S3). At the critical configuration, the tip of the $\mathbf{G}_{m,n}$ is on the inner sphere of the Ewald shell, i.e. the $|k_2\text{-}k_1|$ sphere. Then the scattered FW wave vector $\mathbf{k}_1$' must be in the direction normal to the $|k_2\text{-}k_1|$ sphere. So the value of β is determined by Eq. (3).

The directions of the vectors vary during the rotation process to reach the annihilation critical position. We now use a last index to inform the position of a vector, before or after the rotation. $\mathbf{k}_{1,1}$ is the wave vector of the FW before the rotation, and $\mathbf{k}_{1,2}$ after. $\mathbf{G}_{m,n,1}$ and $\mathbf{G}_{m,n,2}$ are the RLVs with the similar implication (Fig. S4). We assume that after the rotation, the $\mathbf{G}_{m,n,2}$ and $\mathbf{k}_{1,2}$ are in their critical configuration. From Fig. S4, one can conclude that $\theta+\gamma=\theta_k+\beta$, which is exactly Eq. (2) suggests, $\theta-\theta_k=-(\gamma-\beta)$.

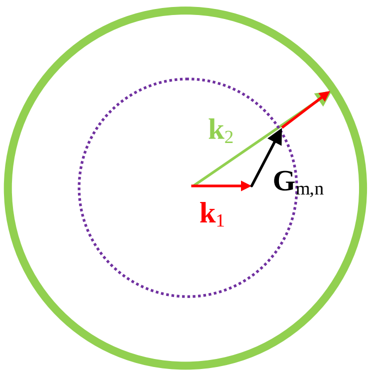

FIG S3: Critical configuration of the conical beam related to the RLV $\mathbf{G}_{m,n}$.

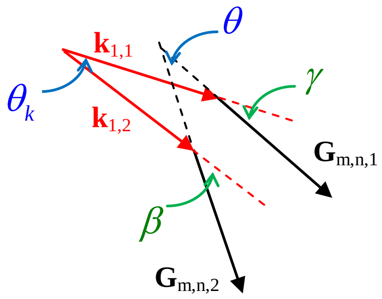

FIG S4: The rotation of the relative vectors during an annihilation process.

Experimental setup

Here we provide the scheme of our experimental setup in Fig. S5. We hope it's helpful to our readers.

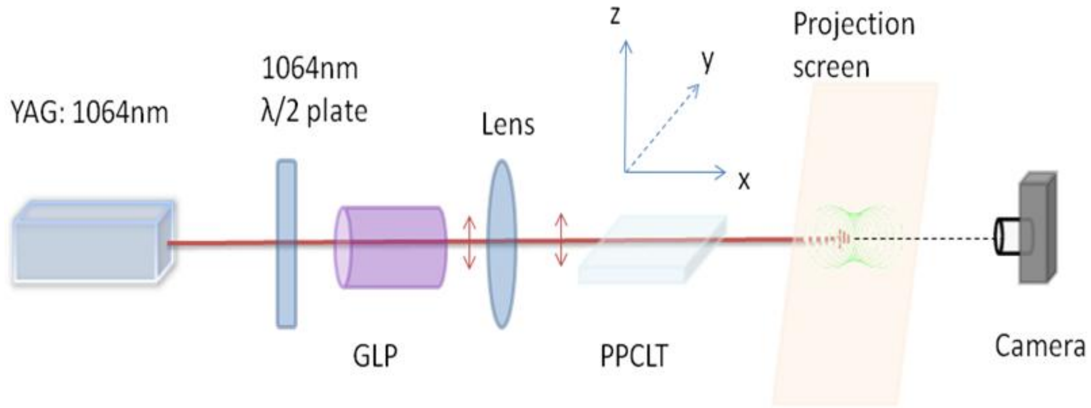

FIG S5: Experimental setup. To simplify the illustration, we don't present the black box that was used to hold the sample and the rotating multi-dimensional adjustment platform under the sample here.

Real-time Ewald shell example

Fig. S6 shows the actual Ewald shell we use in the analysis of our experiment. Parameters and equations used to acquire this figure includes: the period along the x-axis & y-axis Px=12.15μm, Py=12.61μm, FW is a 1064nm infrared laser, experimental temperature is at room temperature 25°, and the dispersion relation follows

$$n_e^2(\lambda,T) = 4.514261 + \frac{0.011901+1.82194\times10^{-8}T^2}{\lambda^2-[0.110744+1.5662\times10^{-8}T^2]^2} + \frac{0.076144}{\lambda^2-0.195596^2} - 0.02323\lambda^2$$

(Nikogosyan D. N. $LiTaO_3$, Lithium Tantalate (LT). *Nonlinear Optical Crystals: A Complete Survey*. Springer Science+Business Media, Inc. (2005).)

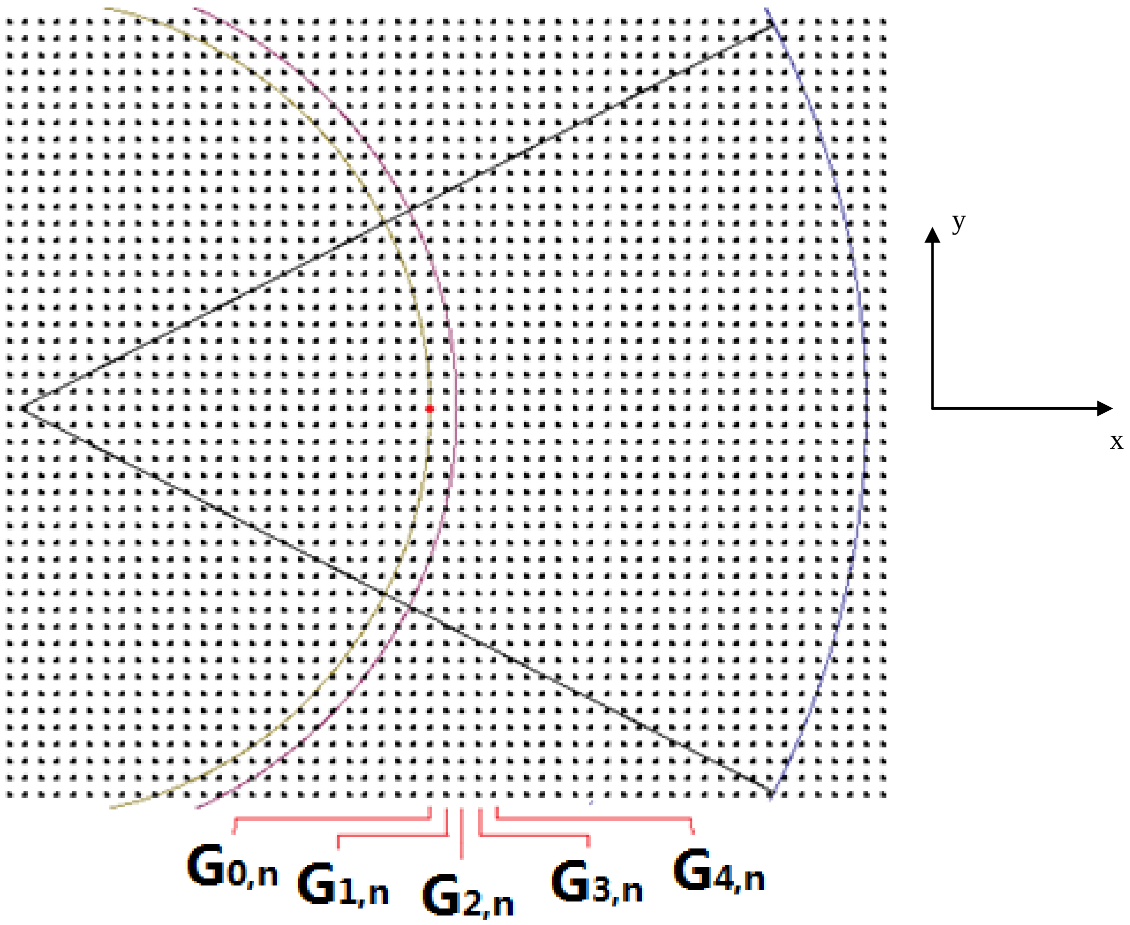

FIG S6: An actual example of the Ewald shell (symmetrical z-cut). The three circular arcs, from left to right, are with diameters of k_1, $|k_2-k_1|$ and k_2, respectively. The red spot on the middle point of k_1 arc is the origin of the reciprocal space O. Each column of the black dots along the y-axis is a series of $\mathbf{G}_{m,n}$ reciprocal lattice point. We explicitly point out a few, namely m=0,1,2,3,4 series of $\mathbf{G}_{m,n}$. A pair of additional lines is drawn from the centre of the Ewald shell onto the k_2 arc. These two lines indicate the direction of the SH critical angle in the xy-plane. Only a small portion of RLVs related to these points in the shell will take effect and their related conical beams be detected. The less deviation the RLV is from the $\mathbf{k}_1$, the easier its conical beam could be detected.
